# Erzhi Pill^®^ Protected Experimental Liver Injury Against Apoptosis via the PI3K/Akt/Raptor/Rictor Pathway

**DOI:** 10.3389/fphar.2018.00283

**Published:** 2018-03-27

**Authors:** Hai-Mei Zhao, Xiao-Yun Zhang, Xiu-Yun Lu, Song-Ren Yu, Xin Wang, Yong Zou, Zheng-Yun Zuo, Duan-Yong Liu, Bu-Gao Zhou

**Affiliations:** ^1^School of Basic Medical Sciences, Jiangxi University of Traditional Chinese Medicine, Nanchang, China; ^2^Department of Postgraduate, Jiangxi University of Traditional Chinese Medicine, Nanchang, China; ^3^Science and Technology College, Jiangxi University of Traditional Chinese Medicine, Nanchang, China; ^4^Editorial Department, Jiangxi University of Traditional Chinese Medicine, Nanchang, China; ^5^Xiangyang Hospital of Traditional Chinese Medicine, Xiangyang, China; ^6^Affiliated Hospital of Jiangxi University of Traditional Chinese Medicine, Nanchang, China

**Keywords:** Erzhi Pill, PI3K/Akt, Raptor, Rictor, apoptosis

## Abstract

Erzhi Pill (EZP) is one of the basic prescriptions for treating liver diseases in traditional Chinese medicine. However, its mechanism of action is still undefined. The PI3K/AKT/Raptor/Rictor signaling pathway is closely related to apoptosis and plays a significant role in the pathogenesis of liver disease. To define the mechanism of the hepatoprotective effect of EZP in the treatment of liver disease, hepatic injury induced by 2-acetylaminofluorene/partial hepatectomy was treated by EZP for 14 days. The therapeutic effect of EZP was confirmed by the decreased production of aspartate aminotransferase and alanine aminotransferase, recovery of pathological liver injury, followed by inhibition of pro-inflammatory cytokines and transforming growth factor-β1. Bromodeoxyuridine assay and TUNEL staining indicated that apoptosis was suppressed and the numbers of cells in S phase and G0/G1phase were decreased. The crucial proteins in the PI3K/AKT/Raptor/Rictor signaling pathway were deactivated in rats with experimental liver injury treated by EZP. These results indicated that the hepatoprotective effect of EZP via inhibition of hepatocyte apoptosis was closely related to repression of the PI3K/Akt/Raptor/Rictor signaling pathway.

## Introduction

Chronic liver disease (CLD), including autoimmune hepatitis, primary biliary cirrhosis, primary sclerosing cholangitis, hepatocellular carcinoma, alcoholic liver disease, and non-alcoholic fatty liver disease, is common in China, and is one of the most important causes of death worldwide, with 1.03 million deaths per year (Lozano et al., 2012). The etiology of CLD remains partially clear. The pathological and serological characteristics of CLD are elevated aminotransferase levels, inflammation, steatosis, hepatocyte apoptosis/death, irregular hepatocyte regeneration, and fibrous tissue hyperplasia (Xi et al., 2016). Perpetual liver injury repeatedly develops and leads to hepatic fibrosis and cirrhosis with liver dysfunction and portal hypertension to rapid deterioration. Conventional methods are utilized to treat CLD, including anti-inflammatory, antiviral and immunoregulatory agents, adjustment of lipidemia, modulation of intestinal flora and symptomatic treatment (Czaja, 2014; Mitchell et al., 2016). However, the development of modern drugs is limited by adverse effects, low patient tolerance and high recurrence rate (Teschke and Danan, 2017). In China, the multitudinous prescriptions of traditional Chinese medicine for CLD are universally accepted by many Chinese people because of their well-deserved therapeutic effect and few adverse effects. These prescriptions include Erzhi Pill (EZP) and Shugan-Huayu powder (Cheng et al., 2010; Jing et al., 2016).

Erzhi Pill is a frequently used prescription for CLD, and is composed of *Fructus Ligustri Lucidi* and *Herba Ecliptae*, which are the basic components of hepatoprotective prescriptions. Treatment of experimental hepatic injury with the active ingredients of EZP has demonstrated the protective effect of EZP on the pathology of acute liver damage. Many studies have shown that the different extracts of EZP have an obvious hepatoprotective effect, which include recovery of hepatic function, decreased serum enzyme activity [alanine aminotransferase (ALT), aspartate aminotransferase (AST) and alcohol dehydrogenase], antioxidants (superoxide dismutase and malondialdehyde), and pro-inflammatory factors [tumor necrosis factor-α (TNF-α), interferon-γ and interleukin (IL)-18] to rectify hepatic damage in CC1_4_-, H_2_O_2_- and ethanol-induced experimental liver injury (Cai et al., 2011; Yan et al., 2012; Nie and Yao, 2014). However, the hepatoprotective mechanism of EZP remains unclear.

It is known that excessive hepatocellular apoptosis is a prominent pathological feature of liver diseases caused by alcohol, viruses, toxic bile acids, fatty acids, drugs, and immune response. In the histopathology of liver disease, including acute and chronic hepatitis, apoptosis plays a significant role in the course of hepatocyte death. Hepatic apoptosis induces many forms of pathological liver injury including liver dysfunction, fibrosis/cirrhosis, and tumorigenesis. Thus, the balance between apoptotic and anti-apoptotic activity plays a pivotal role in the progression of liver disease, and is considered to be a target for treatment of liver disease (Wang, 2014, 2015). Hepatic apoptosis is controlled by multiple signaling pathways such as PI3K/AKT, nuclear factor, TNF receptor (TNFR), Fas/Fas L (Fas ligand), Bax and TRAIL (TNF-related apoptosis-inducing ligand) (Wang and Lin, 2012).

The PI3K/AKT/Raptor/Rictor signaling pathway is one of the major signaling pathways associated with various cellular processes that include cell proliferation, differentiation and apoptosis by activating cell membrane receptor tyrosine kinases in the nucleus (Porta and Figlin, 2009; Brzezianska and Pastuszak-Lewandoska, 2011). Activation of the signaling pathway exhibits multiple biological functions involved in the regulation of autophagy and the cell cycle (Davis et al., 2014; Duan et al., 2014; Heras-Sandoval et al., 2014; Liu et al., 2014). Excessive autophagy and accelerated cell cycle induced cell apoptosis and death (Zhao et al., 2013). The PI3K/AKT/Raptor/Rictor pathway is being explored as a target in the development of liver disease (Rodon et al., 2013).

However, it is unclear whether EZP inhibits the PI3K/AKT/Raptor/Rictor signaling pathway to treat liver diseases. In our previous study, we had demonstrated that EZP relieved liver injury induced by 2-acetylaminofluorene/partial hepatectomy (2-AAF/PH), and decreased aminotransferase levels to protect liver tissues by deactivating the TSC/mTOR signaling pathway (Zhou et al., 2017). Nevertheless, as the upstream signal proteins of the TSC/mTOR signaling pathway, the change in the PI3K/AKT signaling pathway and cell cycle were not observed. In the present study, we investigated further the histological character of hepatocyte apoptosis, cell cycle, and activation of the PI3K/AKT/Raptor/Rictor pathway to definite the mechanism of the hepatoprotective effect of EZP.

## Materials and Methods

### Animals

Male Wistar rats weighing 180–220 g were obtained from the Animal Center of Peking University Health Science Center (animal certificate number SCXK 2006-0008). These animals were in-house bred and maintained on standard laboratory chow and daily 12-h light/dark cycles. The rats were maintained under constant room temperature (25°C) and provided with free access to a standard diet and tap water in accordance with institutional guidelines. The rats were acclimatized to these conditions for 7 days prior to any experimental studies. They were handled in accordance with the guidelines on animal welfare according to the Institutional Animal Care and Use Committee (IACUC) of Jiangxi University of Traditional Chinese Medicine. The protocol (permit number: JZ2015-036) of the present study was approved by IACUC. Forty rats were randomly distributed into five groups: normal (Normal), 2-AAF/PH model (Model), 2-AAF/PH rats treated with EZP by prophylactic administration (EZPP), 2-AAF/PH rats treated with EZP by therapeutic administration (EZPT), and 2-AAF/PH rats treated with rapamycin (RAPA). Each group comprised eight rats.

### Drugs

Erzhi Pill (batch no. Z32020882) was produced by Lei Yun Shang Pharmaceutical Company (Chifeng, China). 2-AAF was purchased from Sigma (St. Louis, MO, United States). Rapamycin (batch no. 20150483) was from Wyeth Pharmaceuticals Company (Philadelphia, PA, United States).

### Liver Injury Induction by 2-AAF/PH

All surgical procedures were performed under sodium pentobarbital anesthesia. Except for rats in the normal group, rats in the 2-AAF/PH model (2-AAF/PH) were treated according to the procedure described by Shiota et al. (2000) and Oh et al. (2007). Briefly, 15 mg/kg of 2-AAF was administered daily by gavage for 7 days. A standard one-third PH was performed 7 days after starting 2-AAF administration. Experimental procedures were approved by the Animal Care Committee of Jiangxi University of Traditional Chinese Medicine and performed in compliance with the guidelines of the university. The animals received human care according to the criteria outlined in the *Guide for the Care and Use of Laboratory Animals* prepared by the National Academy of Sciences. In the normal group, the abdomens of rats were opened, and 1 mL whole blood was taken from the portal vein.

### Treatment Protocol

2-Acetylaminofluorene/partial hepatectomy was administered in the model and the other three treatment groups, and rats in the EZPP group were orally administered 6.48 g/kg EZP until they were killed. However, on the second day after PH, rats in the EZPT and RAPA groups were, respectively, administered 6.48 g/kg EZP and 0.2 mg/kg RAPA by gavage for 14 days. The rats in the normal and model groups were orally administered the same volume of physiological saline. All administrations were performed until the rats were killed. After anesthesia with 10% urethane, the rats were killed on day 14 after PH.

### Assessment of Liver Function

Peripheral blood (*n* = 8) was collected from the aorta ventralis in rats anesthetized with urethane, and separated into serum. The serum was analyzed using an automatic biochemical analyzer. The main indexes of liver function included albumin, ALT and AST.

### Hematoxylin and Eosin (HE) Staining

After the rats (*n* = 8) were killed, the liver was removed and fixed in 10% neutral buffered formalin, embedded in paraffin, sectioned at 3-mm thickness, and stained with HE. Histological observations were made by light microscopy.

### Tunnel Assay

TUNEL staining was performed with an Apop Tag Fluorescein *in situ* Apoptosis Detection kit from Millipore (Boston, MA, United States). Liver tissues were fixed in 4% paraformaldehyde for 7 days and embedded in paraffin. Before TUNEL staining, the sections underwent dewaxing, rehydration in an alcohol gradient, and were thoroughly rinsed with PBS. Afterward the terminal deoxynucleotidyl transferase enzyme mixture was used to treat sections at 37°C for 1 h, and kept in a humidified chamber without light. These sections were incubated with the anti-digoxigenin antibody conjugated to fluorescein for 30 min in the dark. Finally, these sections were observed with laser confocal microscopy. Negative controls were obtained by omission of terminal deoxynucleotidyl transferase.

### ELISA

Liver tissues (*n* = 8) were lysed in RIPA buffer (50 mM Tris-HCl at pH 7.4, 150 mM sodium chloride, 1% NP-40, 0.5% sodium deoxycholate, and 0.1% sodium dodecyl sulfate) with protease and phosphate inhibitor cocktail (Merck, Ashland, MA, United States) using a sonicator. Crude lysates were centrifuged at 20,000 × *g* for 20 min at 4°C. A part of the supernatant (*n* = 8) was used to measure the level of TNF-α, IL-1β, IL-6 and transforming growth factor (TGF)-β1 using commercial ELISA kits (eBioscience, San Diego, CA, United States). The absorbance at 450 nm was read by a microplate reader (Bio-Rad, Hemel Hempstead, United Kingdom).

### Hepatocyte Isolation

Hepatocytes were isolated by two-step collagenase perfusion of the liver followed by isodensity centrifugation in Percoll as described previously (Kao et al., 1996). Viability was determined by trypan blue exclusion and was >90%.

### Bromodeoxyuridine (BrdU) Staining

Bromodeoxyuridine (BrdU) staining *in vivo* was performed as described previously (Magavi and Macklis, 2008) and BD Pharmingen BrdU Flow Kits instruction manual (No. 557891) (BD Company, San Diego, CA, United States). A 10 mg/mL solution of BrdU in sterile 1× DPBS was provided for *in vivo* use. Mice were injected intraperitoneally with 100–200 μL (1–2 mg) BrdU solution. After 1 h post-injection, the hepatocytes were resuspended and incubated with 100 μL Cytofix/Cytoperm buffer for 30 min at room temperature, and washed with perm/wash buffer. The hepatocytes were incubated with 100 μL Cytoperm Permeabilization buffer for 10 min on ice. After they were washed and treated again with Cytofix/Cytoperm buffer for 5 min at 4°C, they were incubated with 100 μL diluted DNase (diluted to 300 μg/mL in DPBS) for 1 h at 37°C. Resuspended liver cells were incubated with 50 μL FITC-anti-BrdU for 20 min at room temperature. The hepatocytes were incubated with 20 μL 7-AAD in 1 mL staining buffer in the dark, and were analyzed by flow cytometry (FACSCalibur; BD Company).

### Western Blotting

Protein concentrations (*n* = 4) were determined in the supernatant of liver tissues by classic BCA protein assay (Beyotime). Equal amounts of protein from each sample were fractionated by SDS-PAGE and transferred onto polyvinylidene fluoride membranes using Bio-Rad western blotting apparatus. The membranes were blocked with 5% fat-free milk or 5% bovine serum albumin, and then probed with the following primary antibodies for 12 h at 4°C: anti-GAPDH (1:2000), anti-PI3K (1:1000), anti-Akt (1:1000), anti-p-Akt (1:1000), anti-PTEN (1:500), anti-Raptor (1:1000), anti-Rictor (1:800), anti-p-Rb (1:1000), anti-C-fos (1:800), anti-C-myc (1:1000), anti-C-jun (1:1000), anti-Fas (1:1000), and anti-Fas-L (1:1000) (Abcam, Cambridge, United Kingdom). The membranes were incubated with appropriate horseradish-peroxidase-conjugated secondary antibodies (1:2000–1:3000, Abcam), and visualized with an enhanced chemiluminescence (ECL) detection kit (Millipore). Bands were quantified using Quantity One version 4.40 software (Bio-Rad).

### Statistical Analysis

Data were expressed as mean ± standard error of the mean (SEM). The statistical significance was determined using one-way analysis of variance, and performed by Prism version 4.0 software (GraphPad Software, La Jolla, CA, United States). A *p*-value < 0.05 was considered significant.

## Results

### EZP Ameliorated Liver Function in Rats With 2-AAF/PH-Induced Liver Injury

**FIGURE 1:**
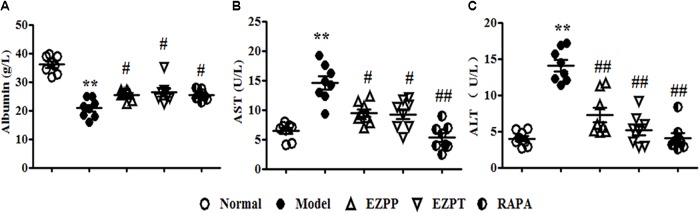
Levels of albumin, AST and ALT in serum. An array of parameter in serum enzyme activity was used to evaluate liver function. These parameters included albumin **(A)**, AST **(B)**, and ALT **(C)**. Data are expressed as mean ± SEM (*n* = 8). ^∗∗^*p* < 0.01 versus Normal group; ^#^*p* < 0.05 and ^##^*p* < 0.01 versus Model group.

Because of 2-AAF-limited hepatocyte cytothesis, liver damage occurred persistently to induce dysfunction of hepatic cells on albumin synthesis and metabolism of various enzymes. In the 2AAF/PH model, secretion of albumin was decreased (**Figure 1A**), while the levels of AST (**Figure 1B**) and ALT (**Figure 1C**) were increased compared with those in the Normal group. However, compared with the 2-AAF/PH model group, the yield of albumin was increased (**Figure 1A**), while the levels of AST (**Figure 1B**) and ALT (**Figure 1C**) were decreased in the 2-AAF/PH rats treated with EZP and RAPA. These results showed that EZP recovered hepatic function to prevent 2-AAF/PH-induced liver injury.

### EZP Attenuated Pathological Injury in Rats With 2-AAF/PH-Induced Liver Injury

**FIGURE 2:**
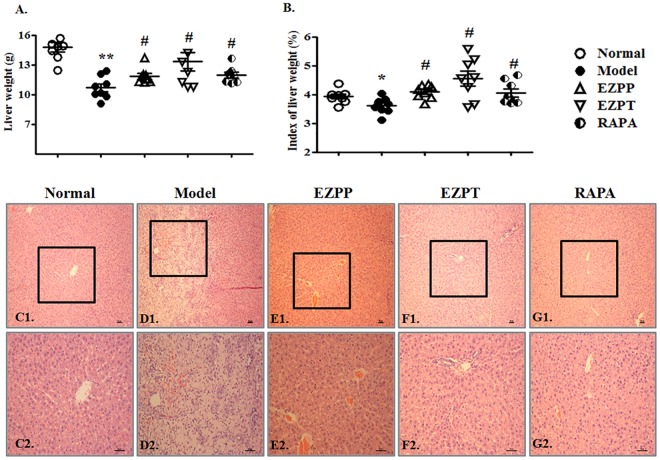
Pathological changes in liver injury induced by 2-AAF/PH. The index of liver weight was measured as liver weight (g)/body weight (g) × 100%. **(A)** Liver weight; **(B)** index of liver weight; liver histology is shown in Normal group **(C1,C2)**, Model group **(D1,D2)**, EZPP group **(E1,E2)**, EZPT group **(F1,F2)**, and RAPA group **(G1,G2)**. Pathology slides of liver were stained with HE. C1, D1, E1, F1, and G1: bar = 100 μm; C2, D2, E2, F2 and G2: bar = 200 μm. Data are expressed as mean ± SEM (*n* = 8). ^∗^*p* < 0.05 and ^∗∗^*p* < 0.01 versus Normal group; ^#^*p* < 0.05 versus Model group.

Although hepatocytes have vigorous vitality, 2AAF-induced disorder of hepatocyte regeneration limited the self-repair ability of the liver and aggravated liver injury, whose main characteristics were weight change and pathological morphology. The liver weight and index of liver weights in 2-AAF/PH-treated rats were lower than those in the Normal, EZPP, EZPT, and RAPA groups (**Figures 2A,B**). There were structural disorder of hepatic lobules, mass hepatocyte necrosis, fibrous tissue proliferation, inflammatory cell infiltration, tissue hyperemia and edema in the liver tissues from rats in the 2-AAF/PH model (**Figures 2C1,C2,D1,D2**). However, in the EZPP (**Figures 2E1,E2**), EZPT (**Figures 2F1,F2**) and RAPA (**Figures 2G1,G2**) groups, similar pathological changes were seen, with intact hepatic lobules, central vein hyperemia, less hepatocyte necrosis, hepatocyte fatty degeneration and/or edema, and irregular newborn hepatocytes as dikaryotic or unequal-sized cells. These results indicated that EZP attenuated the 2-AAF/PH-induced pathological liver injury.

### EZP Inhibited Pro-inflammatory Factors and TGF-β1 Expression in Rats With 2-AAF/PH-Induced Liver Injury

**FIGURE 3:**
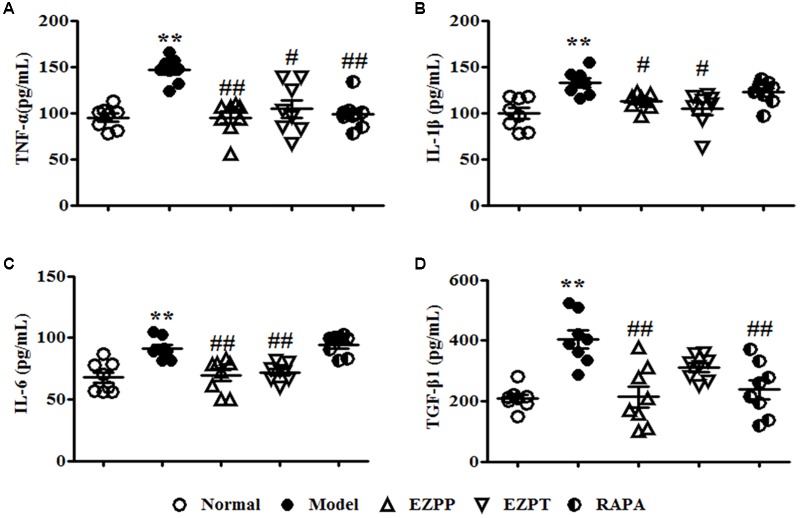
ELISA analysis of related pro-inflammatory cytokines and TGF-β1 in serum. Pro-inflammatory cytokines and TGF-β1 play important roles in the inflammatory reaction and hepatic fibrosis to induce liver diseases. TNF-α **(A)**, IL-1β **(B)**, IL-6 **(C)**, and TGF-β1 **(D)**. Data are expressed as mean ± SEM (*n* = 8). ^∗^*p* < 0.05 and ^∗∗^*p* < 0.01 versus Normal group; ^#^*p* < 0.05 and ^##^*p* < 0.01 versus Model group.

The yields of pro-inflammatory factors (TNF-α, IL-1β, and IL-6) were higher in the 2-AAF/PH model group than in the EZPP, EZPT, and RAPA groups (**Figures 3A–C**). The secretion level of TGF-β1 in peripheral blood in the Model group was increased (**Figure 3D**), but it was decreased in the EZPP and RAPA groups. However, there was little difference between the Model and EZPT groups. The results suggested that EZP inhibited pro-inflammatory factors and TGF-β1 expression in peripheral blood from rats with experimental liver injury.

### EZP Regulated Hepatocyte Apoptosis and Cell Cycle in Rats With 2-AAF/PH-Induced Liver Injury

**FIGURE 4:**
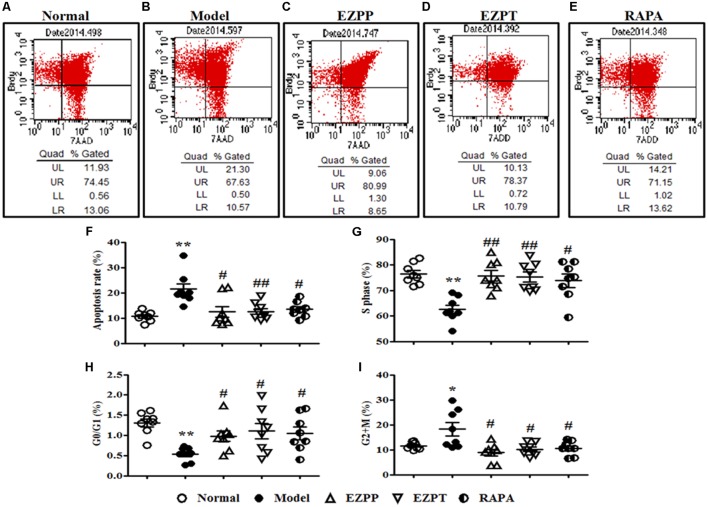
BrdU staining in hepatocytes from rats with liver injury induced by 2AAF/PH. Cellular incorporation of BrdU staining can be detected by flow cytometry to analyze apoptosis and proliferation *in vivo*. BrdU in hepatocyte of rats induced by 2AAF/PHx was labeled by intraperitoneal injection of BrdU, and measured by flow cytometry and staining with anti-BrdU antibody and 7-AAD. The representative scatter diagrams of each group are shown: Normal **(A)**, Model group **(B)**, EZPP group **(C)**, EZPT group **(D)**, and RAPA group **(E)**. Results were analyzed by Flowjo version 7.6.1 software, which included apoptosis **(F)**, and number of cells in S phase **(G)**, G0/G1 phase **(H)** and G2+M phase **(I)**. Data are expressed as mean ± SEM (*n* = 8). ^∗^*p* < 0.05 and ^∗∗^*p* < 0.01 versus Normal group; ^#^*p* < 0.05 and ^##^*p* < 0.01 versus Model group.

Hepatocyte apoptosis and cell cycle were an important index to evaluate survival of liver cells, which were analyzed by flow cytometry (**Figures 4A–F**). Compared with the Normal group, the apoptosis rate of liver cells was increased in the 2-AAF/PH rats without treatment (**Figures 4A,B,F**), and was decreased in the liver injury rats treated with EZP and RAPA (**Figure 4F**). The numbers of liver cells in S phase and G0/G1 phase in the 2-AAF/PH model group were lower than those in the EZPP, EZPT and RAPA groups (**Figures 4G,H**). However, the number of liver cells in G2+M phase was increased in the 2-AAF/PH model rats, and was down-regulated in the EZPP, EZPT and RAPA groups (**Figure 4I**).

**FIGURE 5:**
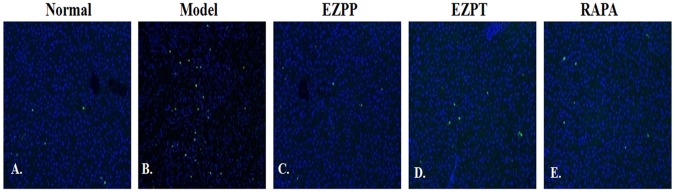
TUNEL staining in liver tissues. TUNEL staining (green) and DAPI (blue)-stained liver sections of rats with liver injury induced by 2AAF/PH. Normal **(A)**, Model group **(B)**, EZPP group **(C)**, EZPT group **(D)**, and RAPA group **(E)**.

Fluorescence microscopy showed TUNEL-positive nuclei in the injured hepatic lobules in the rats with 2-AAF/PH-induced liver injury without treatment, but they were decreased in the rats treated with EZP and RAPA (**Figure 5**).

### EZP Regulated PI3K/Akt/PTEN Signaling Pathway in Liver Tissues

**FIGURE 6:**
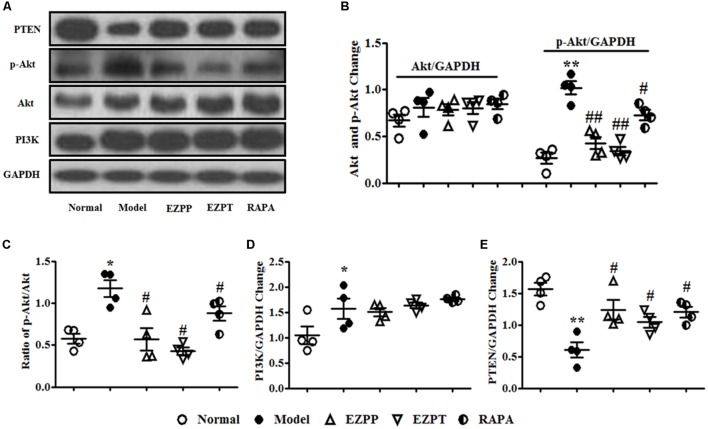
Western blotting of PI3K, Akt, and PTEN. **(A)** Western blotting of PI3K, Akt, p-Akt, and PTEN. **(B)** Quantitative analysis of Akt and p-Akt. **(C)** Ratio of p-Akt/Akt. **(D)** Quantitative analysis of PI3K. **(E)** Quantitative analysis of PTEN. Data are presented as mean ± SEM (*n* = 3). ^∗^*p* < 0.05 and ^∗∗^*p* < 0.01 versus Normal group; ^#^*p* < 0.05 and ^##^*p* < 0.01 versus Model group.

The PI3K/Akt/PTEN signaling pathway plays an important role in regulation of apoptosis. Expression of phosphoinositide 3-kinase (PI3K) and phosphor (p)-Akt proteins was higher in the 2-AAF/PH-induced liver injury rats without treatment compared with the Normal group, which was followed with an increased ratio of p-Akt/Akt (**Figures 6A–D**). Increased levels of p-Akt and p-Akt/Akt ratio were decreased by administration of EZP and RAPA (**Figures 6A–D**). However, the expression of PTEN protein in liver injury rats in the Model group was lower than in the Normal, EZPP, EZPT, and RAPA groups (**Figures 6A,E**).

### Effect of EZP on Expression of Raptor and Rictor in Liver Tissues

**FIGURE 7:**
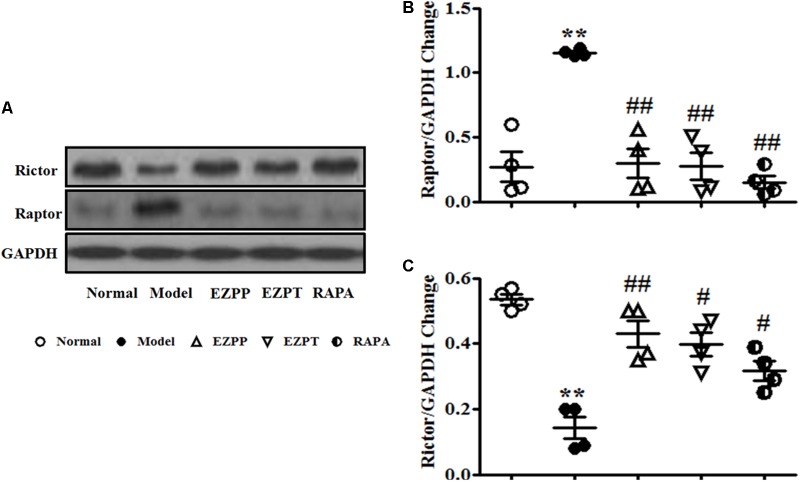
Western blotting of Raptor and Rictor. **(A)** Western blotting of Raptor and Rictor. **(B)** Quantitative analysis of Raptor. **(C)** Quantitative analysis of Rictor. Data are presented as mean ± SEM (*n* = 3). ^∗∗^*p* < 0.01 versus Normal group; ^#^*p* < 0.05 and ^##^*p* < 0.01 versus Model group.

Raptor and Rictor are two main downstream proteins of the PI3K/Akt pathway. Increased expression of Raptor and decreased Rictor expression were found in liver tissues of rats in the 2-AAF/PH model group (**Figures 7A–C**). Compared with the Model group, Raptor protein expression was inhibited, and the level of Rictor was increased after liver injury rats were treated with EZP and RAPA (**Figures 7A–C**).

### EZP Suppresses Expression of Apoptosis Regulatory Factors in Liver Tissues

**FIGURE 8:**
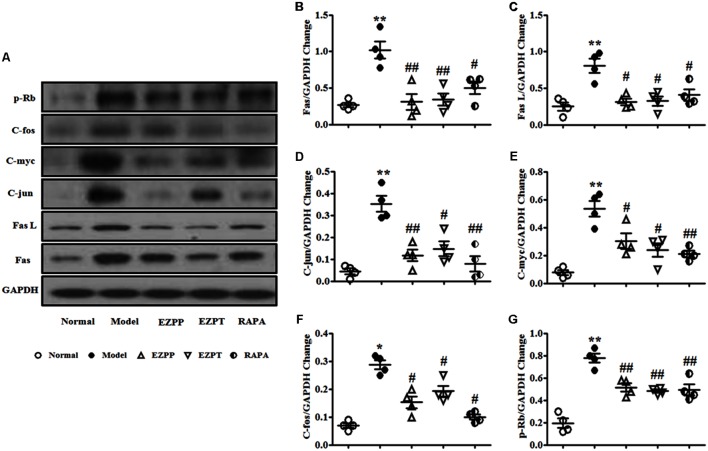
Western blotting of Fas, Fas L, C-jun, C-myc, C-fos, and p-Rb. **(A)** Western blotting of Fas, Fas L, C-jun, C-myc, C-fos, and p-Rb. **(B)** Quantitative analysis of Fas. **(C)** Quantitative analysis of Fas L. **(D)** Quantitative analysis of C-jun. **(E)** Quantitative analysis of C-myc. **(F)** Quantitative analysis of C-fos. **(G)** Quantitative analysis of p-Rb. Data are presented as mean ± SEM (*n* = 3). ^∗^*p* < 0.05 and ^∗∗^*p* < 0.01 versus Normal group; ^#^*p* < 0.05 and ^##^*p* < 0.01 versus Model group.

Apoptosis regulatory factors are key factors in the control of apoptosis, including Fas, Fas L, C-jun, C-fos, C-myc and retinoblastoma protein (p-Rb). Expression of Fas (**Figures 8A,B**), Fas L (**Figures 8A,C**), C-jun (**Figures 8A,D**), C-myc (**Figures 8A,E**), C-fos (**Figures 8A,F**) and p-Rb (**Figures 8A,G**) was elevated in the rats with 2-AAF/PH-induced liver injury without treatment when they were compared with the Normal group. Expression of these apoptosis regulatory factors was suppressed after treatment with EZP and RAPA (**Figure 8**).

## Discussion

The characteristics of liver injury recurred in the rats with 2-AAF-induced liver injury without treatment, which included decreased concentration of albumin, and elevated AST and ALT, and typical pathological changes. The changes in the Model group were consistent with those in our previous studies (Zhou et al., 2017). We showed that 2-AAF/PH-induced hepatic injury was successfully prepared in the present study. We still found that excessive hepatocyte apoptosis was a well-synchronized and common pathophysiological feature of liver injury induced by 2-AAF/PH, as in our previous study, which stimulated inflammation and resulted in the production of TGF-β1 and proinflammatory cytokines (TNF-α, IL-1β and IL-6), further fuelling the inflammatory and fibrogenic reaction. It is known that these changes are closely related to progression of liver disease (Canbay et al., 2003; Feldstein et al., 2003; Grivennikov et al., 2010).

There is mounting evidence to suggest that directly targeting hepatocyte apoptosis to inhibit inflammatory injury of liver disease may be a viable therapeutic strategy. The remedial and prophylactic administration of EZP should be a useful therapeutic strategy for treatment of experimental liver injury. BrdU analysis and TUNEL staining both showed that EZP inhibited the extent of hepatocyte apoptosis, and decreased production of pro-inflammatory cytokines and TGF-β1, liver injuries were alleviated, and the levels of ALT and AST were decreased. These results suggest that the protective effect of EZP is realized by inhibition of hepatocyte apoptosis.

As a pattern of cell death, apoptosis is a complex and essential physiological process that is controlled by many signaling pathways, such as PI3K/AKT, Fas/Fas, Bax/BCL, nuclear factor and TNFR (Taylor et al., 2008). The PI3K/AKT signaling pathway is crucial in autophagy, cell metabolism, cell survival, and cell death in response to various stimuli (Rodon et al., 2013). PI3K can activate Akt protein expression, and in turn phosphorylate and activate mechanistic target of rapamycin complex 1 (mTORC1) or mTORC2, which are composed of mTOR, Raptor and Rictor, however, phosphatase and tensin homolog (PTEN) can inhibit the PI3K/Akt pathway to promote autophagy (Yu et al., 2010; Dibble and Cantley, 2015; Zhang et al., 2015). Activated PI3K/Akt pathway subsequently stimulates extracellular signal-regulated kinase, p38 and mitogen-activated protein kinase to induce Raptor (mTORC1) activation. Over-expression of Raptor activates the downstream molecules, including eukaryotic translation initiation factor 4E-binding protein 1 and p70s6k, to inhibit Rictor (mTORC2), while Raptor and its downstream proteins increase expression of C-myc, C-jun, C-fos p-Rb and other apoptosis-related proteins. These activated apoptosis-related proteins accelerate G1/S cell cycle transition and result in apoptosis and cell death (Iiboshi et al., 1999; Ryu and Han, 2011). In the present study, the apoptosis rate and the percentage of cells in G2 phase, and the number of cells in the S and G0/G1 phase were increased in the experimental liver injury rats, which were confirmed by TUNEL staining. Western blotting showed that expression of p-AKT, PI3K, Raptor, Fas, Fas L, and apoptosis-related genes (C-jun, C-mys, C-fos, and p-Rb) was increased, while the levels of PTEN and Rictor were reduced. These results indicate that excessive hepatocyte apoptosis induces cell death and releases abundant pro-inflammatory factors to induce pathological liver injury. Therefore, we think that the activated axis of PI3K/Akt-Raptor/Rictor plays a pivotal role in the pathogenesis of hepatic injury induced by 2AAF/PH. We also suggest that the axis of PI3K/Akt-Raptor/Rictor is a potential pathway to treat various liver diseases. After administration of EZP, followed by decreased hepatic apoptosis, the levels of PI3K, p-Akt Raptor, C-jun, C-mys, C-fos, and p-Rb were reduced, while the expressions of Rictor and PTEN were increased. This suggests that EZP directly inhibits activation of PI3K/Akt or indirectly decreases Akt phosphorylation by high expression of PTEN or Rictor, and then suppresses Raptor to decrease expression of apoptosis-related genes, and to accelerate cell cycle transition, and finally inhibits excess hepatic apoptosis. EZP is a complex system composed of multitudinous effective components, for example, salidroside and oleanolic acid, which have multiple pharmacological actions, including hepatoprotective, anti-apoptotic, anti-inflammatory, and immunoregulatory activity (Gao et al., 2010). A lot of evidence indicates that salidroside inhibits neurocyte apoptosis to protect against Parkinson’s disease, and that it reduces brain cell apoptosis to improve long-term behavioral and histological outcomes that are realized by suppression of phosphorylation of PI3K, Akt, and mTOR (Chen et al., 2012; Fan et al., 2016; Zhang et al., 2016). Oleanolic acid is another important component of EZP and is widely used to treat liver diseases. It has hepatoprotective, anti-inflammatory and anti-cancer activity, and improves histological status and decreases serum ALT and IL-1β in the treatment of hepatic ischemia–reperfusion injury. Oleanolic acid also suppresses the PI3K/Akt/mTOR signaling pathway to inhibit cell survival and proliferation of prostate cancer cells (Gui et al., 2014; Li et al., 2016; Shi et al., 2016). EZP exerts a hepatoprotective effect against 2AAF/PHx-induced hepatic injury by inhibiting hepatocyte apoptosis, and its active component may suppress the PI3K/Akt/Raptor/Rictor signaling pathway. However, its mechanism of action and therapeutic target are ambiguous.

In recent years, following the intensive research on PI3K/mTOR, nucleotide-binding domain, leucine-rich repeat containing proteins (NLRs) are found and become a special focus, which can regulate inflammatory response, cell proliferation and death, immune response and gut microbiot. The dysregulation of the functional activity of NLRs leads to autoimmune disease including IBD. Especially the NLRC3, Karki et al. (2016, 2017) had indicated that NLRC3 blocked activation of the PI3K-dependent kinase AKT following engagement of growth factor receptors or TLR, or inhibited expression of c-myc, FoxO3a and FoxO1, and suppressed the activation of the mTOR signaling pathway to protect against colorectal cancer. Their study had unveiled that NLRC3 may be a key inhibitor of PI3K/mTOR signaling pathway (Karki et al., 2016, 2017). So these hypothesis was proposed that EZP can improve the expression of NLRC3 to block the activation of PI3K/mTOR signaling pathway to protect liver, and its mechanism will be verified via genomics and molecular biological technique in our next work. NLRC3/PI3K will be anticipated as a worthy target spot to EZP prevented and treated CLD.

## Conclusion

Erzhi Pill inhibits hepatocyte apoptosis to alleviate experimental liver injury and improve liver function by repressing the PI3K/Akt/Raptor/Rictor signaling pathway.

## Author Contributions

D-YL and B-GZ designed the research. H-MZ, X-YZ, X-YL, S-RY, XW, and YZ performed the research. Z-YZ and D-YL contributed the new reagents and analytic tools. H-MZ, Z-YZ, B-GZ, and D-YL analyzed the data. H-MZ and D-YL wrote the paper.

## Conflict of Interest Statement

The authors declare that the research was conducted in the absence of any commercial or financial relationships that could be construed as a potential conflict of interest.
